# Multisensory maps in parietal cortex^☆^

**DOI:** 10.1016/j.conb.2013.08.014

**Published:** 2014-02

**Authors:** Martin I Sereno, Ruey-Song Huang

**Affiliations:** 1 Cognitive Perceptual and Brain Sciences, University College London, London, UK; 2 Department of Psychological Sciences, Birkbeck/UCL Centre for NeuroImaging (BUCNI), Birkbeck College, University of London, London, UK; 3 Institute for Neural Computation, University of California San Diego, La Jolla, CA, United States

## Abstract

Parietal cortex has long been known to be a site of sensorimotor integration. Recent findings in humans have shown that it is divided up into a number of small areas somewhat specialized for eye movements, reaching, and hand movements, but also face-related movements (avoidance, eating), lower body movements, and movements coordinating multiple body parts. The majority of these areas contain rough sensory (receptotopic) maps, including a substantial multisensory representation of the lower body and lower visual field immediately medial to face VIP. There is strong evidence for retinotopic remapping in LIP and face-centered remapping in VIP, and weaker evidence for hand-centered remapping. The larger size of the functionally distinct inferior parietal default mode network in humans compared to monkeys results in a superior and medial displacement of middle parietal areas (e.g., the saccade-related LIP's). Multisensory superior parietal areas located anterior to the angular gyrus such as AIP and VIP are less medially displaced relative to macaque monkeys, so that human LIP paradoxically ends up medial to human VIP.

## Highlights

• A new parietal multisensory area integrates lower body and lower visual field.
• Rearrangement of parietal areas in human and non-human primates is rationalized.
• *In vivo* myelin mapping outlines some parietal multisensory areas.
• Multisensory parietal areas transform visual maps into non-retinocentric coordinates.

**Current Opinion in Neurobiology** 2014, **24**:39–46

This review comes from a themed issue on **Neural maps**

Edited by **David Fitzpatrick** and **Nachum Ulanovsky**

For a complete overview see the Issue and the Editorial

Available online 2nd October 2013

0959-4388/$ – see front matter, © 2013 The Authors. Published by Elsevier Ltd. All rights reserved.

**http://dx.doi.org/10.1016/j.conb.2013.08.014**

## Unisensory versus multisensory

The shortest path between any pair of neurons in the brain often involves just few intervening synapses. For example, in mice, primary visual cortex projects directly to entorhinal cortex [1^••^]; similarly, in primates, parietal visual areas project directly, if sparsely, to V1 [2,3^••^]. Thus, in some sense, every brain area is potentially a ‘multisensory’ area [4,5].

But taking primate V1 as an example, single-unit spikes there are most strongly modulated by the presence of simple visual features (orientation, direction of movement) in the classical excitatory receptive field, or by large arrays of similar low-level visual features in the non-classical surround. Simple auditory, vestibular, and somatosensory stimuli have small effects on the spiking of primate V1 neurons, though they can more strongly modulate the size or latency of subthreshold membrane potentials, and consequently EEG/MEG or fMRI signals. By contrast, spiking activity in neurons in an explicitly multisensory area, such as primate ventral parietal area (VIP) and rodent rostrolateral area (RL), is typically strongly modulated by both visual and somatosensory stimuli applied to localized regions of the receptor sheets, either individually or in combination.

Another consideration is that species differ in the overall depth of their visual cortical area hierarchies. For example, in small nocturnal mammals that have less well developed visual capabilities, like mice, V1 neurons are more strongly modulated by the behavioral context of stimuli (e.g., see [6]); in primates, there are more intervening synapses from motor cortex to V1 [1^••^,3^••^], which might explain why primate V1 is more strictly visual at the level of single units. This review concentrates on mapping overtly multisensory areas in parietal cortex (for previous reviews, see [7–9,10^••^,11]).

## Ventral intraparietal area (VIP) — the parietal face area

VIP was originally defined in macaque monkeys as a visual area containing neurons with large visual receptive fields that also had aligned somatosensory receptive fields on the face and shoulders [12]. More recent experiments have suggested that VIP might instead be thought of as a somatosensory area focused on operations in face-centered space that also has visual input. Avoidance and defensive motor responses from stimulating VIP [10^••^,13] and a preference for stereoscopic stimuli near the face [14] suggest that one primary function is to protect the face.

In humans, a multisensory area containing somatotopic maps of air-puff stimuli to the face superimposed and aligned with retinotopic maps of up-close visual stimuli was found in the postcentral sulcus, just posterior and slightly medial to the S-I hand representation [15,16] in a region originally identified as multisensory by Bremmer *et al*. [17]. This region is also activated during paradigms as diverse as mental arithmetic [18] and delayed reaches in complete darkness toward extinguished visual targets [19], and so it is likely to be involved in many cognitive functions involving actions or events in real or metaphorical peripersonal space. For example, when we say ‘the holidays are approaching’, we treat the holidays as if they were looming objects (compare the syntactically equivalent ‘the children are approaching’) [20]. One of the overlaid functions of multisensory parietal areas in humans may be to generate or interpret the meaning of such utterances.

**Figure 1:**
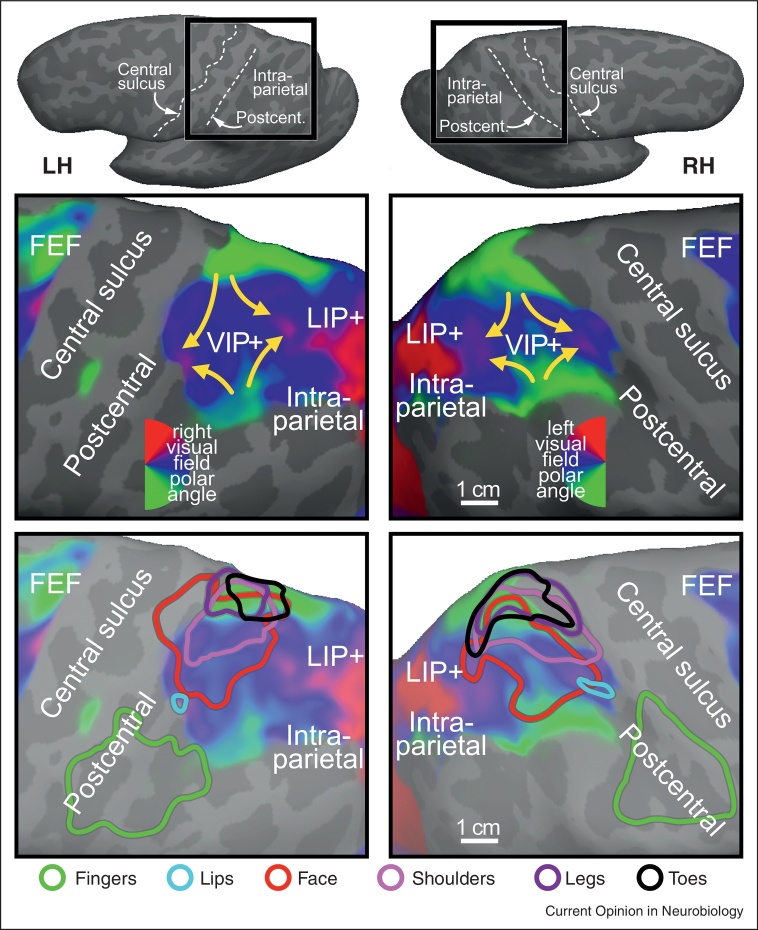
Overlapping retinotopic (upper panel) and somatotopic (lower panel) maps in human anterior parietal cortex. The upper row close-ups show 24-subject average polar angle maps from wide-field direct-view fMRI mapping using a moving video wedge (complex-valued surface-based spherical coordinate system averaging method described in [56]). Four lower → middle → upper field traverses are visible in VIP+ in each hemisphere (yellow arrows). The color contours in the lower row show spherically aligned somatotopic whole body mapping data from [24^••^] (face data [15]) over the grayed visual data (body part key is at bottom). Top insets show the location of the magnified views on the unfolded, dorsolaterally tilted cortex.

Recent fMRI evidence in macaque monkeys has demonstrated that face somatosensory inputs and visual inputs overlap in one or more localized regions of the fundus of the intraparietal sulcus rather than extending along its entire length [21]. This result is compatible with human mapping studies, which have uncovered multiple, somewhat variable, overlapped representations of the face and retina [15]. Surface-based cross-subject average retinotopic maps suggest that the population average pattern in the anterior most part of visual parietal cortex consists of two separated (anterior and posterior) upper field representations and two separated (medial and lateral) lower field representations (see Figure 1, upper). This results in four lower-to-upper visual field progressions. Multiple aligned representations of the face overlay a portion of these visual maps (see red contours in Figure 1, lower).

## The parietal body area (greater VIP)

Electrical stimulation studies in parietal cortex of several different non-human primates using the ‘extended stimulation trains’ method [22] had shown that parietal cortex is involved in generating movements well beyond facial defensive movements [23]. Subsequent bimodal (somatosensory and visual) fMRI mapping experiments in humans [24^••^] then revealed that the multisensory zone in superior parietal cortex is larger than was initially suspected (see cyan/pink/purple/black contours, Figure 1, lower). The rough body homunculus in human parietal cortex is arranged in a different order than the ones in MI and SI (where the face is lateral, the hand is intermediate, and the leg is medial). In human superior parietal cortex, moving lateral to medial, the face and lips in VIP proper are adjoined medially by the shoulders, and then further medially by the lower parts of the body (leg and toes), skipping the hand. The hand, by contrast, is represented ‘out of order’, lateral to the VIP face, in area AIP in the lateral part of the post-central sulcus [16,24^••^], which is situated just posterior to the S-I face representation (Figure 1, bottom, green contour). The visual field map overlying the lower body representation in superior parietal cortex is primarily lower field, as would be expected if part of its function was to defend and coordinate the lower part of the body with respect to visual and somatosensory objects in the lower part of near peripersonal space; for example, when watching your step. Several of these results were prefigured in the excellent review by Rizzolatti *et al*. [11].

## Multisensory areas for visually guided reaching

**Figure 2:**
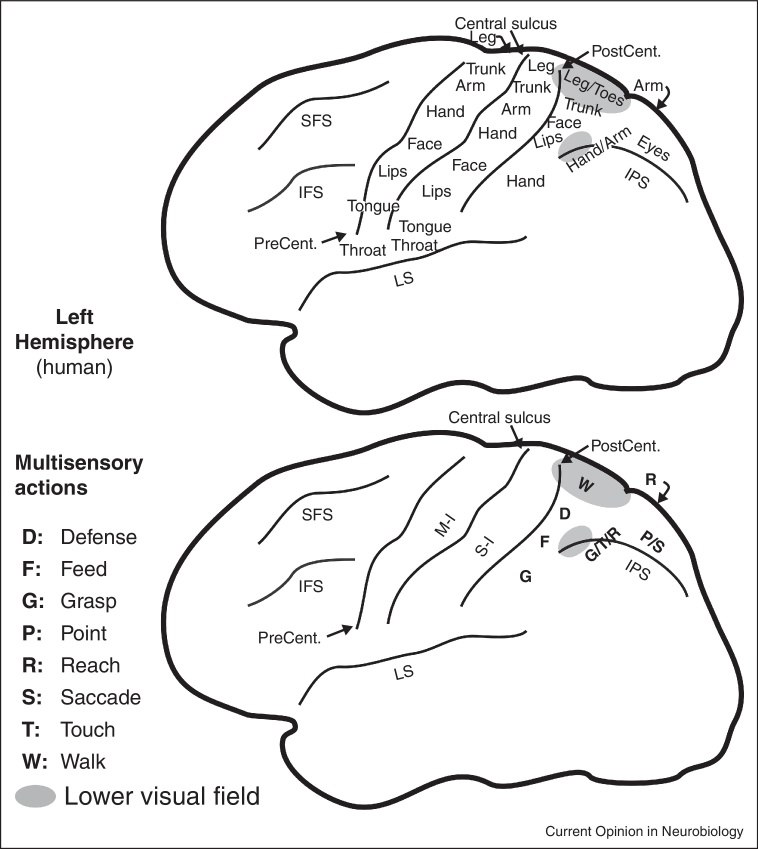
Human parietal eye, hand, face, and body areas. A rough structural (top) and functional (bottom) parcellation of human parietal cortex is shown. Each of the areas is likely to be involved in multiple additional functions beyond those listed here.

There is a separate representation of hand and arm-related multisensory areas more posteriorly on the medial bank of the intraparietal sulcus in macaque monkeys and extending onto the medial wall in the precuneus. This general region has been divided into a number of different areas, some of which overlap each other, including MIP on the lateral surface, PEc near the dorsal convexity, and V6A (itself subdivided), and the greater ‘parietal reach region’ (see [8,25,26,27^••^,28]) on the midline. Recently reach-related and grasp-related areas have been more precisely localized, subdivided, and renamed in humans [29,30,31^••^,32,33,34^••^]. Figure 2 shows a summary of the overall location of body parts (top) and a rough guide to functional localization (bottom) drawn from references [7–9,10^••^,11–16,18,19,21–23,24^••^,25,26,28–30,31^••^,32–33,34^••^].

## Comparative anatomy of parietal areas

**Figure 3:**
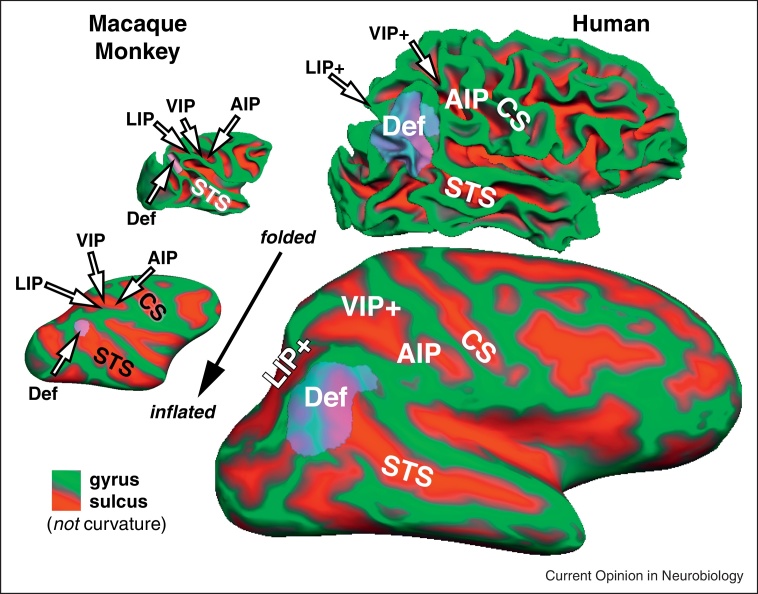
The large relative expansion of the inferior parietal component of the default mode network (Def, transparent purple) in humans compared to macaque monkeys results in the medial displacement of LIP+ (nominally, the lateral intraparietal area) to a position medial to VIP+, shown on folded and inflated macaque and human hemispheres. The monkey parietal default mode network component is taken from [57^••^]; the human angular gyrus default mode network was defined as the zone bounded by retinotopic, tonotopic, and somatotopic maps in this subject. All cortical surfaces are shown at the same scale.

Parietal cortex has long been known to be a site of multisensory interactions on the basis of the effects of brain lesions there in humans and in particular hemi-neglect. Subsequent anatomical and physiological investigations especially in macaque monkeys provided support for this idea. However, some confusion has persisted in correlating invasive non-human primate data with human brain imaging data. In particular, the relatively larger size of the angular gyrus component of the ‘default mode network’ in the inferior parietal lobule of humans compared to macaque monkeys results in a substantial superior and medial displacement of some — but not other — parietal visual areas in humans, compared to their location in non-human primates (see Figure 3). Thus, the initial report of a retinotopically organized human homologue of macaque area LIP was controversial given how close to the midline the putative human LIP was situated [35]. Subsequent studies further subdivided parietal areas on the medial bank of the human IPS [36,37]. The initial unease with such a medial LIP derived from the fact that macaque VIP, in the fundus of the intraparietal sulcus, was conventionally thought of as being medial to LIP, which as LIP's name suggests, sits on the lateral bank of the intraparietal sulcus. But on the unfolded cortex, VIP can also be thought of as anterior to LIP in the sense of being closer to somatosensory cortex. In humans, VIP is mostly anterior to the relatively enlarged angular gyrus areas; this results in VIP being less medially displaced by them than LIP is. This paradoxically results in human VIP+ sitting lateral to human LIP+ (IPS1-4) (see Figure 3).

The superior and medial displacement of human LIP out of the lateral bank of the intraparietal sulcus recalls a related relative displacement of human MT+, which moves laterally and inferiorly out of the superior temporal sulcus, so that like LIP, its position no longer exactly matches its name (the ‘middle temporal area’). That downward displacement is also largely the result of the increased relative size of the lateral parietal default mode network in humans.

Though particularly enlarged in primates, multisensory cortical areas situated in a similar location to VIP seem to be a basic feature of mammalian cortex. For example, in cats, there is the rostral lateral suprasylvian sulcus multisensory area (r-LS, [38]; AESc [39]) that extends medially onto the suprasylvian gyrus; and in rats and mice, there are the V1-recipient and S-I-recipient rostrolateral (RL) and anterior area(A). As in humans and non-human primates, these areas lie at the border between unimodal visual and somatosensory areas, they are anterior and superior to most other extrastriate visual areas, and they are distinctly medial to the representation of the face in primary somatosensory cortex. In cats, area r-LS receives input from motion-sensitive lateral suprasylvian areas just posterior to it as well as registered somatosensory inputs. In rats, area RL contains a mostly lower visual field representation superimposed on a representation of the vibrissae (anatomy and visual mapping from [1^••^], multisensory responses confirmed by Olavarria and Sereno, unpublished observations). It is interesting to note that in contrast to quadrupedal animals, bipeds such as humans have — and may need — a better view of their lower limbs during locomotion, which may partly explain the large size of the human parietal (lower) body area (Figures 1,2).

In a comparative context, it is worth emphasizing that homology does not always imply exact functional similarity. A striking example is that the homologs of the bones that originally formed the articulation of the reptilian jaw (articular, quadrate) are now incorporated into the mammalian middle ear (malleus, incus — still articulated) for impedance-matching between airborne sounds and the fluid filled cochlea. Homologous brain structures often have somewhat different functions, too. For example, in mice, V1 inherits most of its orientation selectivity from surprisingly well-tuned orientation-selective dLGN cells [40], while in cats and primates, orientation selectivity primarily emerges only in V1. Similarly, in cats, neurons in the primary dLGN-recipient layers of V1 are direction-selective, while in primates, strong direction selectivity only emerges one synapse later in non-dLGN-recipient layer 4B. We should therefore not be surprised to see functions jumping one synapse or area forwards or backwards relative to homologous parietal areas in monkeys versus humans.

## Coordinate transformations

Building on the behavioral experiments of Hallet and Lightstone [41], an influential coordinate remapping experiment in the superior colliculus using a double step saccade demonstrated that ‘quasi-visual cells’ (cells that were visual except under these special circumstances) in intermediate collicular layers buffer and then remap the location of extinguished targets in retinotopic coordinates in order to address the correct spatial location in the underlying motor map — a saccade vector map arranged so that each saccade vector underlies an equivalent receptive field center vector [42]. Using similar methods, retinocentric remapping was subsequently shown to occur in macaque LIP [43]. The required eye position signals are relayed to LIP from the frontal eye fields via the dorsomedial thalamus [44], ultimately from the superior colliculus itself.

Data from VIP have suggested that eye position signals are instead used to map visual information into somatosensory (face-centered) coordinates. Coordinate transformations in face VIP are convenient to study there because the face is relatively immobile, the rotatable eyes are in a fixed position near the center of it, and for the most part, the eyes cannot see the face. When eccentric eye position misaligns VIP retinal and somatosensory inputs, a majority of VIP visual receptive fields are partially or fully remapped in the direction of the somatosensory receptive field. By contrast, none of the somatosensory receptive fields are remapped in the direction of the retinal receptive field and instead remain firmly ‘attached’ to the face and shoulders [45].

Functional MRI data have shown that visual signals in VIP in humans are also remapped into somatosensory coordinates. But these studies have also revealed that those head-centered multisensory coordinates were arranged into multiple topological maps arranged in a similar way across subjects (Figure 1) [15,24^••^] — which was not obvious from the single-unit data. It is worth mentioning that fMRI data are coarse-grained since each voxel contains roughly 1 million neurons; invasive recording experiments reveal a more complex underlying picture with some VIP neurons showing only partial visual remapping and a minority not remapping at all [45]. The extent to which visual areas posterior to VIP might also do VIP-like head-centered or body-centered updating has been hotly disputed with a majority arguing that it does not occur there (e.g., [46]).

It is much harder to determine which coordinate system transformations might be occurring with visually guided reaching because the eyes and the limb(s) move independently, and because the eyes see the limb, making it much more difficult to naturalistically control visual stimulation. Recent studies in monkeys and humans [47–49] attempting these difficult manipulations (e.g., using rubber hands to decouple visual and proprioceptive signals) have suggested that a small number of parietal neurons and some visual areas remap visual information into hand-centered coordinates. Critical pathways by which proprioceptive and eye position information gets into parietal cortex are less well understood than is the case with eye movements but may involve the basal ganglia [50].

## Myelin measures in parietal cortex

The myelination of the gray matter varies tangentially between cortical areas, but also with cortical depth and as a function of local curvature of the cortical surface (convex cortical regions are more densely myelinated). By combining newly developed MRI methods for myelin mapping (T_1_/T_2_ ratio [51], quantitative T_1_ estimation [52]) with cortical surface-based and depth-based analysis, it has recently become possible to outline heavily myelinated areas across the entire cortex of single living subjects [51,52]. Heavily myelinated areas have shorter T_1_ relaxation times and are hence brighter in T_1_-weighted images; heavier myelination is therefore positively correlated with relaxation rate, R_1_ (= 1/T_1_).

**Figure 4:**
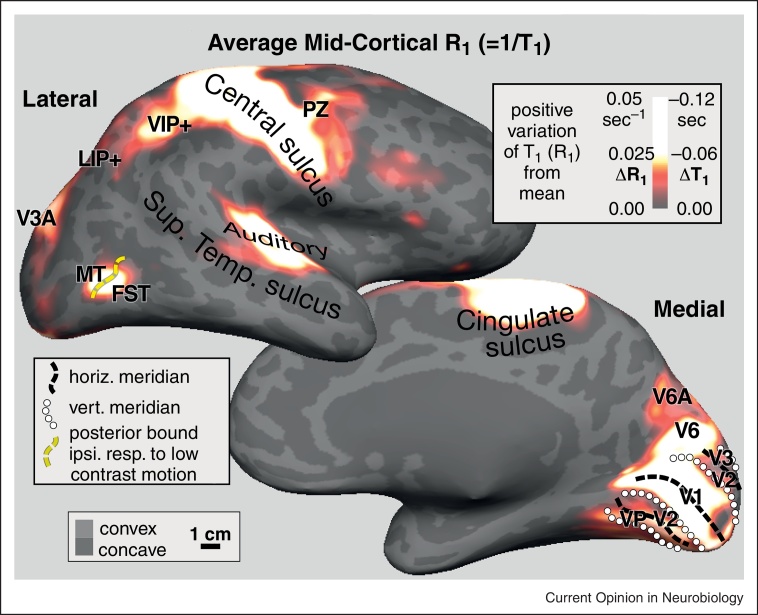
Quantitative relaxation rate (*R*_1_ = 1/*T*_1_) maps demarcate cortical areas with heavy gray matter myelination [51,52]. Spherical morph average maps of quantitative *R*_1_ values sampled at 50% of cortical thickness are illustrated as positive variation from the mean (Δ*R*_1_, maxima shown are 3-4% above mean). As expected, densely myelinated primary visual, auditory, and somatomotor cortex and early visual areas MT/FST, V3A, and V6 have the largest *R*_1_ values. Parietal area VIP is the next most densely myelinated, as is an extension off the motor strip, PZ, the polysensory zone, that responds to passive visual and face somatosensory stimuli. In medial parietal cortex, reach-related area V6A is also myelinated.

In parietal cortex, there is a heavily myelinated region attached to S-I by a small isthmus — and almost as heavily myelinated as S-I (and M-I) — that roughly corresponds to the location of the face and shoulder representation in VIP (based on retinotopy on the same subjects) (see Figure 4). Just posterior to VIP, there is an elongated region of moderately high myelination extending through the LIP/IPS areas that eventually join up with a more prominent maximum in V3A.

Moving medially just beyond the dorsal convexity onto the midline, there is another moderately heavily myelinated zone in a region that has been identified as a human homologue of macaque V6A (note that V6 is even more strongly myelinated than V6A) [34^••^]. This forms the posterior extremity of the human parietal reach region.

Finally, in frontal cortex there is another maximum of myelination in a multisensory motor area identified as PZ [15,16,24^••^,53] — an area strongly interconnected with VIP [54], which appears as an extension off of the M-I motor strip.

These data show a strong resemblance to Flechsig's survey of perinatal infant myelogenesis [55], where he identified not only the heavily myelinated VIP and V6, but also MT and V3A (modern names). Though his work did not receive as much attention as that of Brodmann, in some respects, it more closely matches our current ideas of the parcellation of human neocortex.

## Variability in cortical organization

Normalized cross-subject averaging has a long history in cognitive neuroimaging studies. These methods work best under the assumption that cortical areas in different subjects vary in size but not number or topological relations. Invasive anatomical and electrophysiological mapping experiments in animals, however, suggest that areas vary not only in size but sometimes also in neighbor relations. The same may occur in humans. For example, the number of discrete upper field representations found in individual subjects between the upper field representation of V3A and the more posterior multisensory upper-face-plus-upper-visual-field representation in VIP varies from 1 to 3 in different humans (e.g., see [36]). Given the large differences in individual area size and in neighbor relations among visual areas among closely related primates species, within-species variations are perhaps not surprising.

## Conclusion

Parietal multisensory maps are present in all mammals and are especially well developed in primates and humans. They seem to be specialized for coordinating eye and limb movements in near peripersonal space for the defense of the entire body, but also for acquisitive movements such as hand-to-mouth and biting. In humans, parietal multisensory areas are also active in a variety of cognitive acts, some of which may involve fictive or metaphoric acquisition, object manipulation, or body defense.

Much work remains to be done in the field of active sensory-guided limb movements, which involve complex coordination of sensory inputs (visual, auditory, vestibular, somatosensory) as well as multiple sources of efference copy signals (saccades, smooth eye movements, face and lip movements, neck movements, limb movements, finger and toe movements). This area is particularly challenging because of the difficulty of controlling these multisensory stimuli, and in the case of human neuroimaging, maintaining data quality while making movements.

## References and recommended reading

Papers of particular interest, published within the period of review, have been highlighted as:

• of special interest
•• of outstanding interest
